# Considering the Intercultural Development Inventory (IDI) to Assess Intercultural Competence at U.S. Pharmacy Schools

**DOI:** 10.3390/pharmacy11010039

**Published:** 2023-02-19

**Authors:** Diana Tamer, Yifei Liu, Jennifer Santee

**Affiliations:** Division of Pharmacy Practice and Administration, The University of Missouri–Kansas City School of Pharmacy, Kansas City, MO 64108, USA

**Keywords:** intercultural competence, the intercultural development inventory, validity, health professional education, pharmacy education

## Abstract

Background: U.S. pharmacy schools need to engage in improving intercultural competence among administrators, faculty, staff, and students. The Intercultural Development Inventory (IDI) can be a possible tool to determine the level of intercultural competence. U.S. pharmacy schools need to examine the validity of the IDI within the context of health professional education prior to using this tool. Objectives: To describe the relationship between the IDI and its underlying theory, identify whether the validity of the IDI has been established within two specific contexts, and discuss the practical issues and implications of using the IDI. Methods: Medline, Embase, and selected health professional education journal websites were searched to identify fully published studies utilizing the IDI within health professional education. Eligibility of articles was determined with a standardized approach. Results: Ten studies were identified by full-text reviews, but none investigated the validity of the IDI. Conclusions: The IDI has been shown to be valid in certain contexts, but its validity has yet to be confirmed within health professional education. U.S. pharmacy schools need to examine practical issues and implications when deciding if the resources required to administer, analyze, and report IDI results are reasonable.

## 1. Introduction

A diverse healthcare workforce is essential in reducing the existing health disparities for under-represented minority patients [1,2]. Unfortunately, the proportions of minority pharmacy students in the U.S. are not reflective of the general population [3]. Although minority faculty members have played a major role in encouraging minority students to enroll in pharmacy schools [4], their proportions are also not representative of the U.S. population [5]. In addition, pharmacy students may encounter patients or work with colleagues from different cultures that have cultural practices, products, and perspectives much different than their own [6].

To increase the number of under-represented minority students and faculty members, as well as to prepare pharmacy education for a diverse patient population, U.S. pharmacy schools need to create a more inclusive and culturally responsive environment. This requires engagement in improving intercultural competence to make school administrators, faculty, staff, and students aware of values, beliefs, and norms that are unique to one’s native culture. Effective intercultural communication and relations are centered on intercultural competence, and being adequate in intercultural competence is an important factor to address health disparities.

Intercultural competence can be measured through a variety of assessment tools. In general, there are indirect and direct measures. Intercultural competence can be measured indirectly, such as by using end-of-year school surveys, or directly, such as by using the Intercultural Developmental Inventory (IDI). The IDI is a proprietary self-assessment tool that has been used by institutions. The current version (the third version) is a 50-item instrument that can be completed in 15–20 min [7]. In addition to collecting demographic information, the IDI evaluates seven dimensions: Denial, Polarization (Defense and Reversal), Minimization, Acceptance, Adaptation, and Cultural Disengagement.

The validity of the IDI was established within certain contexts such as recruitment of diverse staff in high-tech organizations and non-health professional students’ study-abroad experience [7]. However, validity in one context may not automatically mean validity in different contexts [8]. To use the IDI at U.S. pharmacy schools, the validity of the IDI within contexts of health professional education would need to be examined. The interpretation of items on the IDI may vary depending on the knowledge and experience of the individual completing the IDI. The knowledge and experience of health professional school administrators, faculty, staff, and students would seem to be quite different from the samples of high-tech organization recruiters and high school students enrolled in a study-abroad experience who were included in initial studies of the IDI’s validity.

This narrative review has three objectives. First, to describe the relationship between the IDI and its underlying theory. Second, to identify if the validity of the IDI has been established within two specific contexts. One context is health professional schools and colleges which provide an inclusive environment for under-represented administrators, faculty, staff, and students. The other context is health professional student performance on other measures of intercultural competence. Third, to discuss practical issues and implications of using the IDI at U.S. pharmacy schools.

## 2. Methods

A literature search for fully published articles was performed from the third week of September to the first week of November 2022. Two online databases available at reviewers’ institution were used—Medline and Embase. “Intercultural development inventory” was chosen as the search term because the IDI is the focus of this review article. For Medline, a search was conducted during the timeframe of 1946 to the third week of September 2022. For Embase, a search was conducted during the timeframe of 1947 to the first week of October 2022. The starting time points of the search periods were the publication dates of the earliest publications in these two databases.

In addition, the search term was used among online search engines of selected journals in the field of either pharmacy education or health professional education. Specifically, the following journals were included: *American Journal of Pharmaceutical Education*, *Currents in Pharmacy Teaching and Learning*, *Journal of the American Pharmaceutical Association*, *Annals of Pharmacotherapy*, *Pharmacotherapy*, *American Journal of Health-System Pharmacy*, *Academic Medicine*, *Medical Teacher*, *Medical Education*, *Perspectives on Medical Education*, *Education Strategies in Medical Sciences*, *Medical Science Educator*, *Postgraduate Medical Journal*, *Academic Emergency Medicine*, *Journal of Professional Nursing*, *Journal of Nursing Education*, *Nurse Education in Practice*, *Nurse Education Today*, *Nurse Educator*, *Nurse Education Perspectives*, *Teaching and Learning in Nursing*, *Journal of Dental Education*, *European Journal of Dental Education*, *Journal of Physician Assistant Education*, *Journal of Physical Therapy Education*, and *Journal of Clinical Education in Physical Therapy*. Reference lists of identified articles were searched as well.

Eligibility of articles was determined with a standardized approach. “In press” articles that were not yet published were not included. One reviewer performed title and abstract reviews to identify studies that utilized the IDI as an outcome measure in health professional education. Two reviewers independently read the full text of identified studies to determine whether any one of the four types of validity was confirmed in the study contexts—face validity, content validity, construct validity, or criterion validity. For instance, whether the investigators had potential subjects talk through what they thought was assessed as they read through the items to confirm face validity, whether the investigators calculated the content validity ratio based on data from subject matter experts to confirm content validity, whether the investigators examined associations between the IDI and other assessments to confirm criterion validity, or whether the investigators performed factor analysis to confirm construct validity. If there was a discrepancy between reviewers, it was discussed in a virtual meeting of reviewers till an agreed resolution. Data collected from full-text reviews included the study subjects, the study countries, the interventions conducted, the comparison groups, and the outcomes.

## 3. Results

The initial search on Medline yielded five articles, and the initial search on Embase yielded 25 articles. From the online search engines of selected journals, the initial search of *American Journal of Pharmaceutical Education* yielded three articles, *Academic Medicine* yielded six articles, *Medical Science Educator* yielded one article, the *Journal of Nursing Education* yielded four articles, *Nurse Education in Practice* yielded two articles, *Nurse Education Today* yielded four articles, *Nurse Educator* yielded one article, and *Journal of Physical Therapy Education* yielded two articles. No articles were found from the other selected journals. The search of identified articles’ reference lists yielded one additional article.

Thirteen publications were under full-text reviews after removing duplicates and reviewing titles and abstracts. Full-text reviews resulted in ten articles that utilized the results from the IDI as an outcome or variable of interest. The remaining three articles did not use the IDI as an outcome. None of the ten articles investigated the validity of the IDI within the two specific contexts. Moreover, none of the studies report IDI results for health professional school administrators, faculty, and staff, while focusing on students. Table 1 summarizes these ten articles.

## 4. Discussion

### 4.1. Theory-Based IDI

The IDI is based on the Developmental Model of Intercultural Sensitivity (DMIS) [19,20,21]. In general, a theory provides the framework, constructs, or outcomes, to test a supported instrument’s face validity, content validity, construct validity, or criterion validity. Therefore, to discuss the validity and practical issues of the IDI, it is important to first understand the relationship between the IDI and the DMIS.

The DMIS consists of six stages—Denial, Defense, Minimization, Acceptance, Adaptation, and Integration, and segments individuals from an ethnocentric orientation to an ethnorelative one [19,20,21]. Denial, Defense, Minimization are three stages of an ethnocentric orientation, whereas Acceptance, Adaptation, Integration are three stages of an ethnorelative orientation. The DMIS implies that individuals’ capabilities of a greater understanding of cultural differences can be learned through stages of progression. Namely, a person can gradually change from an ethnocentric orientation in which one’s own culture is perceived to be more important than other cultures, to an ethnorelative orientation in which other cultures are accepted. Accordingly, interventions can be designed to facilitate individuals to move from one stage to the next stage.

Similarly, the IDI has an intercultural developmental continuum of five stages from Denial to Adaptation, and it indicates two types of mindsets—monocultural and intercultural mindsets [22]. The five stages in IDI are based on the six stages in DMIS. A person in the stage of Denial is unaware that other cultures exist, a person in Defense recognizes different cultures but sees only one culture as superior, a person in Minimization prefers to focus similarities between cultures, a person in Acceptance recognizes and has begun to accept cultural differences, and a person in Adaptation is able to change behaviors and perceptions to match other cultures. Additionally, the two types of mindsets in IDI are consistent with the two orientations in DMIS. A monocultural mindset tends to be ignorant of the background and elements in another culture, which can prevent an individual from interacting with people in that culture effectively. To the opposite, an intercultural mindset is a deep understanding of cultural similarities and differences, which can be learned via interaction with various cultural environments and situations.

Despite the similarities, the IDI differentiates from the DMIS in a few aspects. The IDI has Cultural Disengagement as a separate dimension from the intercultural developmental continuum. Defined as “a sense of disconnection or detachment from a primary cultural group” [22], Cultural Disengagement reflects cultural identity. Moreover, to help users interpret the results, the IDI has developed the IDI Profile along the continuum. For example, within the IDI Profile, Perceived Orientation is self-rated and Developmental Orientation is rated by the IDI, and their difference is known as the Orientation Gap. The IDI also puts forward Trailing and Leading Orientations. The former tends to pull one back from Developmental Orientation, and the latter provides the next step. These differences have strengthened the IDI in its usability and practicality.

### 4.2. Validity of the IDI within Two Specific Contexts

The face validity, content validity, construct validity, and criterion validity of the IDI have been tested appropriately within certain contexts [7]. However, as mentioned before, validity in one context may not transfer to another. In the context of health professional schools and colleges providing an inclusive environment, whether the validity of the IDI holds for health professional school administrators, faculty, staff, and students remains to be demonstrated. In addition, the demographics of the sample providing feedback on clarity and the applicability of response items have not been fully described, leaving it uncertain as to whether health professional school administrators, faculty, and staff would provide similar feedback.

An earlier version of the IDI was developed based on responses of interviewees in the Washington DC area who were born in and outside of the U.S [23]. Interview questions included “Do you think there is much cultural differences around here?” and “What kinds of difficulties or problems associated with having cultural differences around here exist?” [23]. Responses to these questions may differ depending on the community being referenced. An individual is a part of multiple communities with some communities being more diverse than others. For example, an individual’s neighborhood may be relatively homogenous, leading them to not see many cultural differences, while their workplace may be quite diverse. Whether the community was defined for the interviewees is not clear [23], and responses may vary based on interpretation of items. While difficulties due to cultural differences may exist within an individual’s workforce, difficulties due to cultural differences may not exist within an individual’s neighborhood.

The IDI is associated with other assessments which include the ability to hire diverse staff and the knowledge of host culture [7]. However, in the context of health professional students’ performance on other measures of intercultural competence, the IDI hasn’t been reported to correlate with outcomes of interest to schools and colleges of pharmacy. For example, whether schools and colleges provide an inclusive environment for under-represented administrators, faculty, staff, and students, and how well students interact with under-represented patients. In summary, under the two specific contexts that this review aims to explore, little evidence has demonstrated the validity of the IDI.

### 4.3. Practical Issues and Implications

One advantage of using the IDI is that it provides an IDI profile after the responders complete the questionnaire. The IDI profile pinpoints the responder’s or group’s overall position on the development continuum [24]. In particular, the profile reports “trailing” problems that are preventing the responder or group from progressing further along the continuum. Meanwhile, the IDI profile highlights the immediate challenges that the responder or group will encounter. While the opportunities to interact with individuals of diverse backgrounds are growing in U.S. pharmacy schools, the IDI profile can quickly reveal the respondent’s or group’s experience with cultural differences, and thus the profile provides guidance for designing and implementing interventions. However, individual results on the IDI can be different from group results. Halm and colleagues noted for some individuals, when their pre- and post-intervention group orientation was Acceptance, their individual developmental orientation was Minimization [12]. As a self-reported measure, the IDI relies on individuals’ own perceptions of their intercultural experiences, which may not be an accurate reflection. For example, some individuals may be reluctant to admit to areas in which they lack intercultural competence, leading to an overestimation in results.

Nevertheless, when considering the IDI as an assessment tool, some practical issues and implications need to be examined. Several investigators have utilized the IDI as a tool to assess the impact of a particular educational intervention (Table 1). While reviewing the outcomes of these interventions, an emerging theme arose. Most participants at baseline seemed to overestimate their capability in coping with cultural differences and perceived themselves to be in Acceptance prior to interventions. In addition, most studies did not detect a change after the intervention, regardless of how extensive the intervention was (e.g., a few hours vs. more than 20 h). Whether statistically significant but small numerical changes in the IDI scores result in more meaningful outcomes (e.g., patient satisfaction) remains to be seen, limiting the utility of IDI results in determining the efficacy of an intervention, especially when considering interventions that may require extensive time and resources. In addition to what amount of change in the IDI scores signifies a meaningful difference, Punti and Dingle questioned the validity of the IDI and argued that the IDI is not generalizable to Black, Indigenous, and People of Color (BIPOC) [25]. Later, Hammer responded to this critique by pointing out that the interview methodology of Punti and Dingle was flawed [26].

We suggest implementing six steps if a U.S. pharmacy school is interested in using the IDI: (1) estimating the cost of the IDI and receiving approval for its use; (2) deciding what interventions the school wants to survey pre- and post-intervention; (3) training a faculty or staff member to administer the IDI; (4) planning debriefing sessions with participants to explore their results with a trained facilitator and consider growth opportunities; (5) establishing a team or committee to review the results and consider a future for continued improvement; and (6) considering the use of the IDI in conjunction with other assessment measures, such as a patient satisfaction survey and peer evaluation for school administrator, faculty, and staff. Specifically, pharmacy schools should first consider the types of decisions that will be made based on the IDI results, whether they are high-stake or low-stake decisions. On one hand, high-stake decisions (e.g., student progression within the curriculum), especially if based solely on one assessment method such as the IDI, require validity to be firmly established within the context in which that assessment method is used. That is, pharmacy schools would need to confirm validity within their populations when using the IDI in high stakes decisions. On the other hand, low-stake decisions (e.g., determining content of intercultural competency training) based on multiple assessment methods that include the IDI, do not need validity to be as firmly established. Moreover, pharmacy schools need to determine if the resources required to administer, analyze, and report the IDI are worth the utility of the information gathered from the IDI. The utility of IDI results, such as the correlations between changes in scores and outcomes of interest, needs to be investigated.

Many existing tools including the IDI involve self-reported measures. However, the full complexity of intercultural competence is hard to capture with a single tool [27]. Intercultural competence broadly includes effective and appropriate communication and behavior in various intercultural interactions which requires others’ perspectives [28]. It seems vital to use various assessment approaches including both direct and indirect measures to completely assess intercultural competence. Using both types of evidence can also help explain why surveys may show regression when the direct evidence may show progress [27]. Considering more than one tool may be an approach to address the complexity of measuring different aspects of intercultural competence. For instance, the Inventory for Assessing the Process of Cultural Competence among healthcare professionals–Student Version© was used along with the IDI at a U.S. Doctor of Physical Therapy program [29]. Of note, this study was not included in Table 1 because it was an in-press corrected proof at the time of the literature search rather than a published article. This study noted that the healthcare-specific measuring tool, when compared to a more general tool such as the IDI, captured different aspects of cultural competence [29]. For example, while some students were in Minimization or even Acceptance, they did not have the adequate knowledge, skills, and values to operate with cultural proficiency in a healthcare setting. Furthermore, this study highlighted the importance of (1) using multiple measures for assessment to decrease possible bias and increase generalizability and (2) having qualitative and quantitative assessment measures such as self-reflections and feedback from patients or peers. Using multiple measures might help explain why IDI scores improved from pre- to post-intervention period for some studies in Table 1.

At U.S. pharmacy schools, intercultural competence can be formed by various activities. An intercultural experience, such as a study-abroad program or a case-based teaching method based on cross-cultural interaction, may increase students’ knowledge of cultural values, beliefs, and norms. Similarly, an intercultural training session given by a consultant may increase the knowledge among school administrators, faculty, and staff. However, gaining the knowledge is only the beginning of the journey. What is more important is to develop intercultural competence throughout a curriculum, an experiential education, or one’s career with a focus on practicing the knowledge. Namely, how to approach and interact with others from different cultural backgrounds, how to adapt one’s own behavior and communication style according to cultural differences, or how to use active listening and avoid stereotyping. Overall, it must be recognized that the assessment of intercultural competence should be a continuous process instead of a one-time effort, due to its dynamic and complex nature [27].

## 5. Conclusions

Developing intercultural competence is becoming increasingly important in health professional education including pharmacy education. Built on the DMIS, the IDI is a direct measure that can be helpful to gain a global understanding of both the participants’ and the school’s intercultural competence, design an intervention, and assess the impact of such an intervention. Its face validity, content validity, construct validity, and criterion validity have been well established within certain contexts. However, according to our knowledge, its validity has not been reported within health professional education. Although the IDI may offer valuable insights as a measuring tool, practical issues should be considered and results may need to be correlated with additional tools such as patient satisfaction or peer evaluation. In particular, when deciding if the resources required to administer, analyze, and report the IDI results are reasonable. Lastly, demonstrating effective work with patients, the ability to provide culturally responsive health care, and an inclusive environment for historically marginalized students, faculty, and staff seems to require more than one tool to measure intercultural competence. These tools, including the IDI, can be used to initiate discussion and identify areas of growth.

## Figures and Tables

**Table 1:** Summary of studies identified as reporting IDI results.

Study	Subjects	Country	Intervention/Comparison Groups	Whether Change in IDI Score Was Statistically Significant	Whether Stage Change Occurred	Stage of Most Subjects
Altshuler et al. (2003) [9]	Pediatric medical residents	United States	2-h workshop & OSCE; OSCE alone	No No	No No	Minimization Minimization
Boggis (2012) [10]	Occupational therapy students	United States	Occupational therapy students; Other health professional students	No Yes	No Yes	Minimization Baseline: Minimization End: Defense
Fitzgerald et al. (2018) [11]	Nursing students	United States	7-week class & 12-day service learning in Nicaragua; No comparator group	Scores not compared	No	Minimization
Halm et al. (2012) [12]	Nurse, social worker, chaplain, physician, respiratory therapist, nutritionist, pharmacist	United States	Inservice & critical reflection; No comparator group	Scores not compared	No	Minimization
Harder (2018) [13]	Nursing students	Canada	3 brief video vignettes; No comparator group	Scores improved but statistical analysis not completed	No	Minimization
Huckabee et al. (2012) [14]	Physician assistant students	United States	15 contact hours of didactic and experiential learning & 12 months of experiential learning; No comparator group	Students only took the IDI after intervention	Not assessed	Minimization
Kirby et al. (2021) [15]	Nursing students	United States	20 h of didactic learning; No comparator group	Yes	No	Minimization
Kruse et al. (2014) [16]	College of nursing faculty, staff, and students	United States	Students: presented with individual with aggregate and individual IDI results; Faculty, staff: presented with aggregate results and 3-h session on “developing intercultural understanding”; No comparator group	Students only took the IDI before intervention	Not assessed	Minimization
Peiying et al. (2012) [17]	Physical, occupational and speech therapy students	Australia	4-week study abroad experience; No comparator group	No	No	Minimization
Zazzi (2020) [18]	Nursing students	Switzerland	Whole nursing curriculum (specific details of intercultural development training not provided); No comparator group	Yes	No	Minimization

OSCE = observed structural clinical exam.

## Data Availability

Data supporting reported results can be found in Table 1.
